# Pheochromocytoma manifesting as cortical blindness secondary to PRES with associated TMA: a case report and literature review

**DOI:** 10.1186/s12902-022-01109-0

**Published:** 2022-08-15

**Authors:** Sankalp P. Patel, Medjine Jarbath, Lauren Saravis, Peter Senada, David H. Lindner, Robert A. Grossman, Ricardo A. Francosadud

**Affiliations:** 1 Department of Graduate Medical Education, Internal Medicine Residency, NCH Healthcare System, Naples, FL 311 9th St. N34102 USA; 2 Department of Pulmonary/Critical Care Medicine, Associate Program Director of Pulmonary/Critical Care Fellowship, NCH Healthcare System, Naples, USA; 3 Department of General Surgery, General Surgeon, NCH Healthcare System, Naples, USA; 4 Department of Graduate Medical Education, Associate Program Director of Internal Medicine Residency, NCH Healthcare System, Naples, USA

**Keywords:** Pheochromocytoma, Neuroendocrine tumor, Cortical blindness, PRES, Endocrinopathy, Hypertension, Adrenal, TMA

## Abstract

**Background:**

Pheochromocytomas are neoplasms originating from neuroectodermal chromaffin cells leading to excess catecholamine production. They are notorious for causing a triad of headaches, palpitations, and sweats. Though the Menard triad is one to be vigilant of, symptomatic presentation can vary immensely, hence the tumor earning the label “the great masquerader.”

**Case presentation:**

We report a case of pheochromocytoma initially presenting with cortical blindness secondary to posterior reversible encephalopathy syndrome and thrombotic microangiopathy from malignant hypertension. Our patient was seen in our facility less than a week prior to this manifestation and discharged after an unremarkable coronary ischemia work-up. In the outpatient setting, she had been prescribed multiple anti-hypertensives with remarkably elevated blood pressure throughout her hospitalization history.

**Conclusion:**

Pheochromocytoma presenting with malignant hypertension and hypertensive encephalopathy should be expected if left untreated; nonetheless, the precipitation of cortical blindness is rare in the literature. This case contributes an additional vignette to the growing literature revolving adrenal tumors and their symptomatic presentation along with complex management. It also serves to promote increased diagnostic suspicion among clinicians upon evaluating patients with refractory hypertension.

## Background

Pheochromocytomas are rare medullary adrenal tumors, manifesting in the setting of a genetic predisposition or spontaneously [1]. The architecture of the adrenal gland was first described by Eustachius circa 1563, in a study titled *Opscula Anatomica*. Over three centuries later, Felix Frankel described the first medullary adrenal tumor in an 18-year-old woman in 1886; a few decades later, Ludwig Pick coined the term pheochromocytoma in 1912 [2]. Though discovered over a century ago, as many as 50% of pheochromocytomas are described in autopsy patients, simply due to neuroendocrine tumor not being on the differential diagnosis [3]. With advancements in biochemical laboratory testing, imaging studies, and genetic testing, the increased detectability of these tumors provides reassurance for the future. Though categorized as benign tumors in most occurrences, pheochromocytomas often manifest clinically with sustained refractory hypertension, severe headaches, palpitations, and uncontrolled sweating. In addition, their heavily variable symptomatic presentation can falsely be attributed to more common disorders, rightfully earning this tumor the label “the great masquerader” [4]. Previously thought to be incidentally rare, pheochromocytomas and sympathetic paragangliomas have notably increased in incidence over the last half-century almost 5-fold [5].

## Case presentation

A 64-year-old female patient with history of refractory hypertension, prediabetes, and chronic kidney disease (CKD) stage IIIa initially presented to our facility with substernal chest pressure. Upon undergoing exercise stress testing, she exhibited adequate tolerance capacity with unremarkable electrocardiographic (ECG) findings not concerning for ischemia. Concurrently while undergoing exercise stress testing, she suffered a fifth metatarsal fracture and was advised to follow-up in the outpatient setting with orthopedic surgery. She was discharged with a new prescription for metoprolol.

Upon returning home, the patient described three days of refractory nausea and vomiting prompting her to return to the hospital. Her vital signs on arrival were notable for 219/124 mmHg blood pressure, tachycardia with heart rate 117, tachypnea with respiratory rate of 28, SpO2 98% on room air, and afebrile. Physical exam findings on arrival revealed unremarkable ocular examination with pupils equal and reactive to light bilaterally and intact extraocular movements, no thyromegaly, tachycardia with an early-peaking 2/6 systolic ejection murmur best appreciated at the right upper sternal border with bounding peripheral pulses, chronic venous stasis changes, no flank tenderness, intact cranial nerves, and no rashes visualized. Given fluctuating, paroxysmal blood pressures along with continued nausea, the admitting clinician suspected sepsis and admitted the patient to a cardiac telemetry floor.

**Fig. 1:**
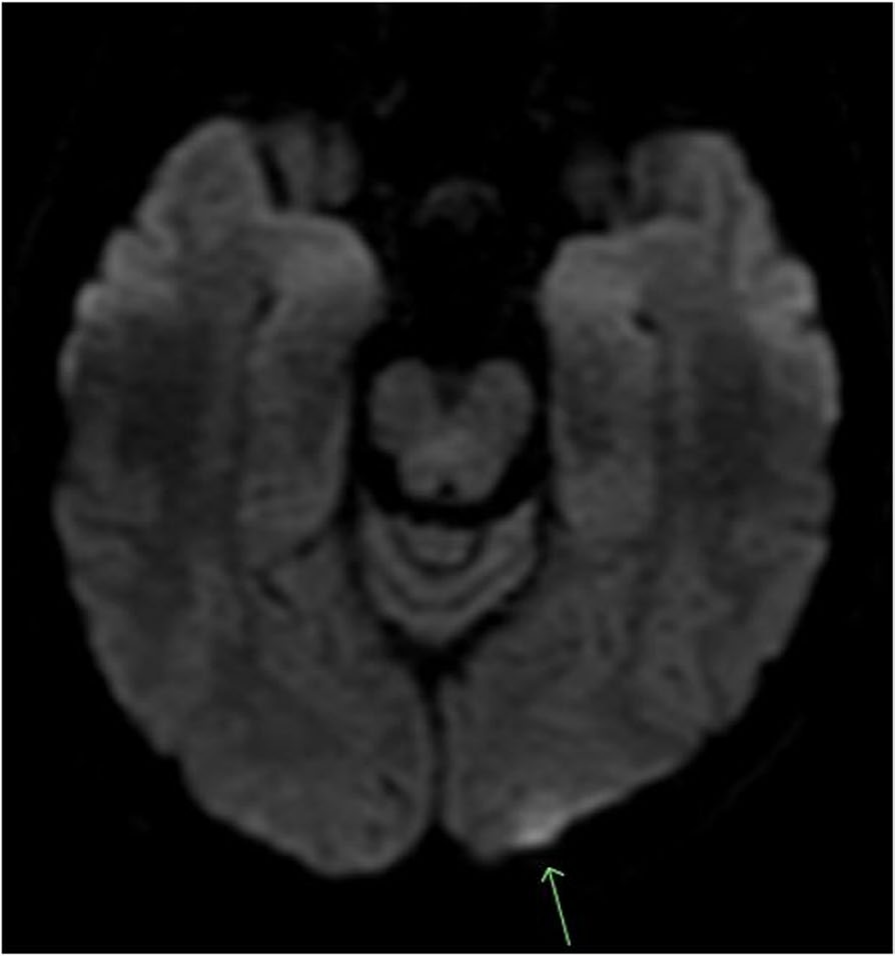
MRI Brain DWI revealing left cortical hyperintensity consistent with acute infarct at watershed zone

Less than a day into admission, she experienced sudden blurry vision and confusion prompting a non-contrast CT scan of her head; a subsequent CTA of the head and neck were also performed revealing ischemic penumbra in both posterior and occipital lobes; the carotids were unremarkable. Thereafter, her blurry vision worsened, and she attested to bilateral cortical blindness; she was taken for MRI of the brain showing bilateral subcortical white matter infarcts, left greater than right, and an acute left occipital cortical infarct in the watershed zone (Fig. 1). Given concomitant AKI on CKD (Table 1), she underwent bilateral renal ultrasound detailing what was read as a large right renal cyst. Coinciding with her deterioration hemodynamically and clinically, her hemoglobin continued to decrease, dropping from over 15 g/dL on admission to 9.6 g/dL within 72 h. The anemia was correlated with a peripheral blood smear revealing 3 + schistocytes consistent with her markedly elevated blood pressures presumed to be due to hypertensive thrombotic microangiopathy. Direct Coomb’s testing was performed and was negative, pointing likely to a hypertensive etiology. Her troponin, originally elevated at 0.35 ng/mL on arrival, peaked to 3.2 ng/mL, and she complained of waxing and waning chest pressure, at which point cardiology was consulted. The patient had been in the hospital less than a week prior undergoing coronary ischemic evaluation and underwent transthoracic echocardiogram revealing hyperdynamic ejection fraction of 70–75%, grade ¼ diastolic dysfunction, without significant valvular pathology or evidence of shunting. A repeat echocardiogram performed during the current admission which revealed no significant changes.

**Table 1:**
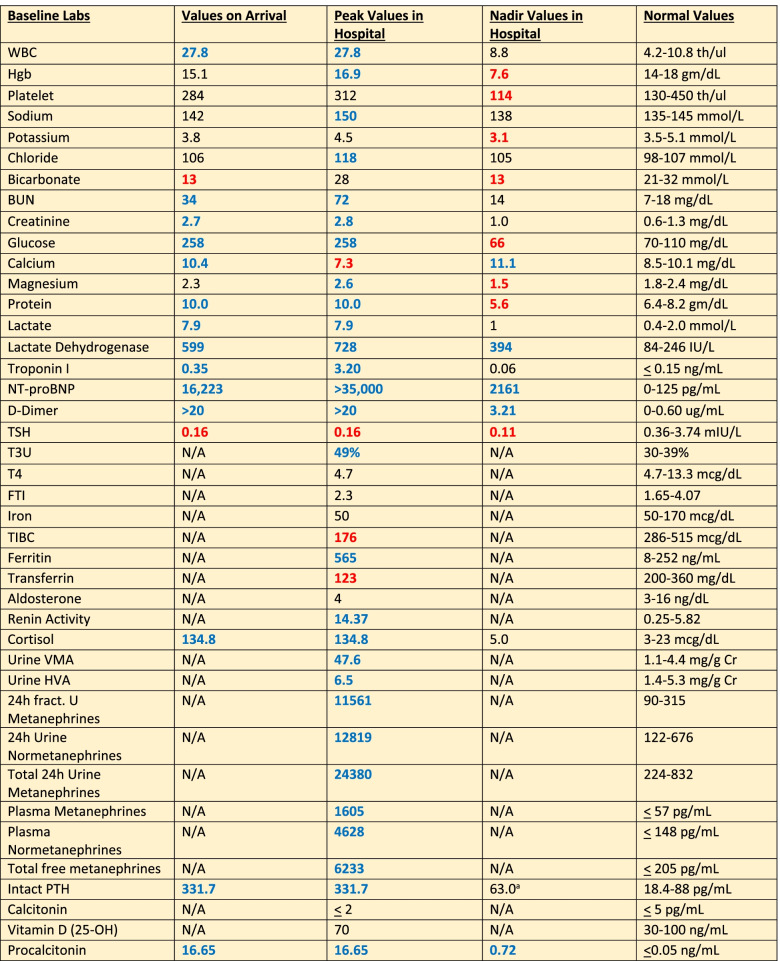
Pertinent Laboratory Studies

Note that blue-colored text reveals elevated values whereas red-colored text denotes decreased levels. An ^“a”^ next to a value stands for a value measured very recently prior to current hospitalization from prior hospitalization.

**Fig. 2:**
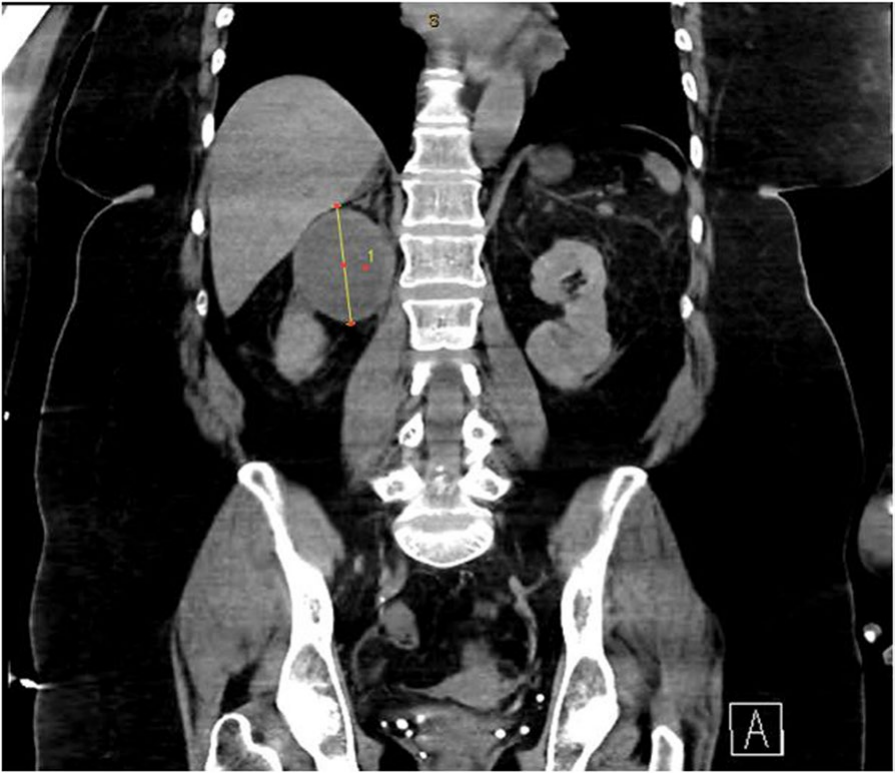
Coronal Slice of CT Abdomen revealing 6.6 m diameter right-sided adrenal mass

**Fig. 3:**
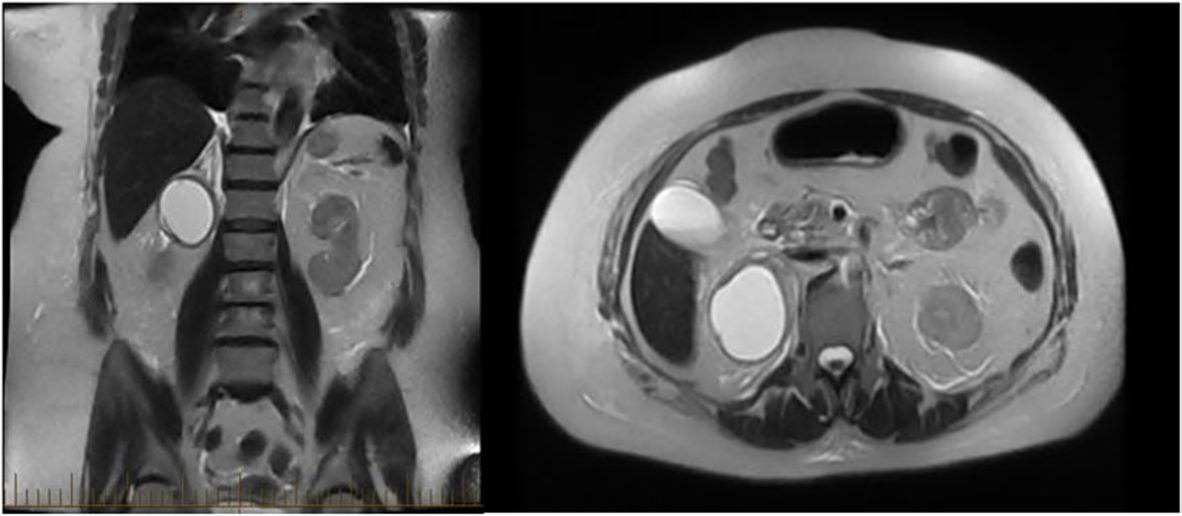
Coronal T2 view of 6.7 cm cystic right adrenal mass on MRI (left) with axial slice on right

**Fig. 4:**
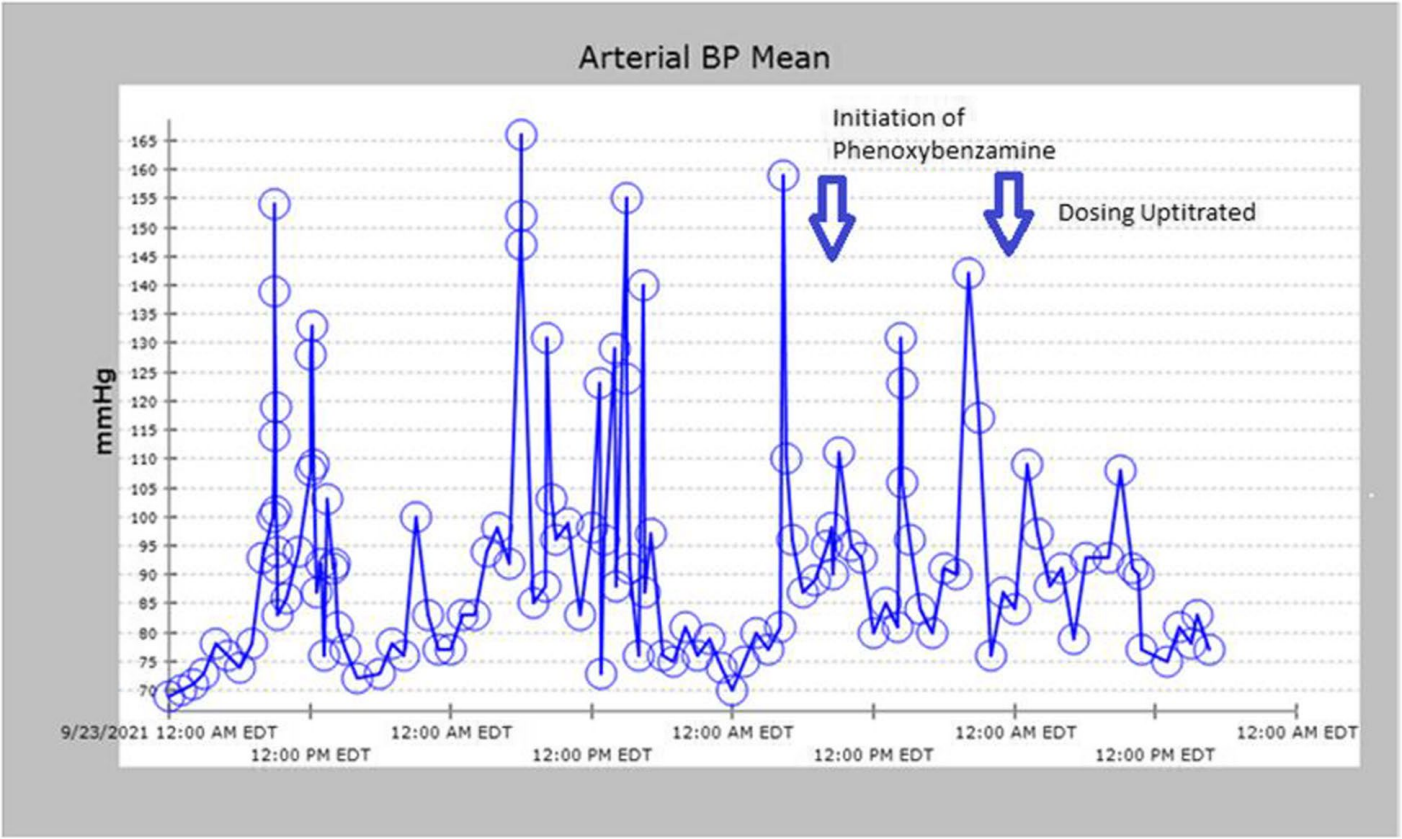
Graph Revealing volatile MAP; see effect of phenoxybenzamine when initiated on 9/25 AM and dose increased on 9/26

Given persistent nausea and vomiting, paroxysmal fluctuant pressure, severe headache now accompanied with bilateral vision loss, and overall decompensation, a CT scan of the abdomen was performed revealing a 66 mm mass in the right suprarenal fossa (Fig. 2). Plasma metanephrines along with an MRI of the Abdomen were ordered, due to suspicion for underlying pheochromocytoma. The MRI confirmed the presence of a 6.7 cm cystic right-sided adrenal mass (Fig. 3). Serum metanephrines, urine metanephrines, and urine VMA/HVA all revealed marked elevation (Table 1). Beta-blockade therapy was immediately stopped, and phenoxybenzamine therapy was initiated, which promptly reduced the patient’s mean arterial pressure (Fig. 4). The dose of phenoxybenzamine was titrated up until the patient reached a maximum dosage of 60 mg twice daily; during this process, general surgery was consulted, and the decision was made to allow for full alpha-blockade preoperatively, with a plan for tumor extirpation. Throughout this time, the patient was closely observed in the intensive care unit, occasionally requiring clevidipine infusion and nitroglycerin infusions to combat elevated blood pressures. Her admission calcium was found to be slightly elevated as well, prompting further evaluation for Multiple Endocrine Neoplasia Syndrome Type 2A. This led to subsequent checking of calcitonin and intact parathyroid hormones, of which the former was within normal range, but the latter was elevated (Table 1) at 331.7 pg/mL. Revisiting her CTA of the neck, multiple small hypodense thyroid nodules were, thus a complete thyroid panel (Table 1) along with thyroid ultrasound were obtained. Thyroid ultrasound showed a mildly asymmetric thyroid gland with very small hypoechoic lesions seen bilaterally, with follow-up recommended in one year. The remainder of her stay until anticipated surgery was quite uneventful, and she noted marked clinical improvement while receiving phenoxybenzamine therapy. Other etiologies for adrenal masses were also excluded during this time through checking aldosterone and renin activity and conferring with cortisol levels showing marked elevation (Table 1). Rheumatologic panel and urine protein electrophoresis were also performed, both of which yielded negative. Two days prior to procedure, an MIBG scintigraphy scan was conducted to ensure that presence of extra-adrenal paragangliomas was excluded, and a detailed visual of the tumor was mapped.

**Fig. 5:**
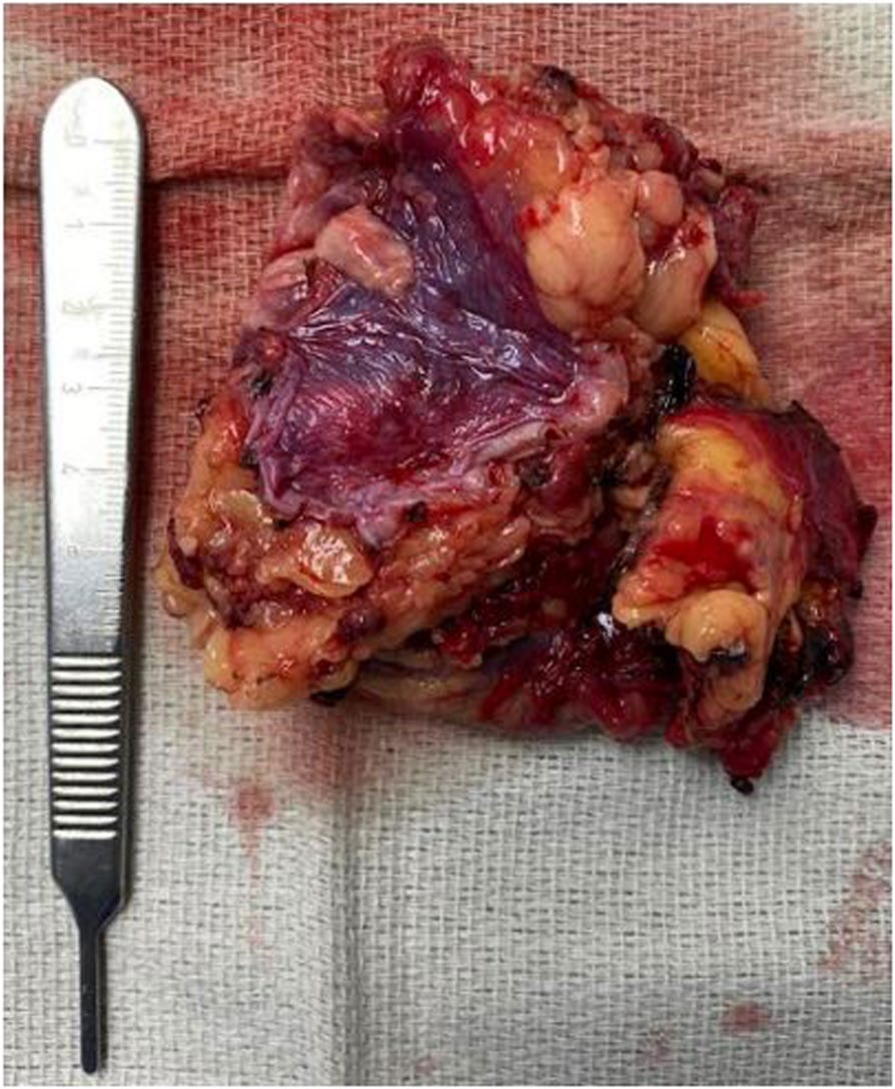
Resected 6.7 cm cystic adrenal mass extending from adrenal medulla believed to be pheochromocytoma

Finally, the anticipated day of surgery arrived, and our patient was taken to the operating room with an expected robotic-assisted laparoscopic adrenalectomy to be performed. Detailed pre-operative planning was conducted through the coordination of anesthesiology, surgery, medicine, and endocrinology collaboration. Pre-operative planning involved anticipated need for significant volume resuscitation throughout the procedure given sudden catecholamine loss leading to profound hypotension; this in junction with concomitant intravenous corticosteroids (100 mg hydrocortisone given intra-operatively) was planned. The patient was planned to return to the intensive care unit for vigilant post-operative monitoring. Given extent of tumor size and complex anatomical variation in our patient with proximity of mass to inferior vena cava, as well as the patient’s obesity, the planned minimally invasive procedure was converted to an open adrenalectomy. No post-operative complications occurred, and the tumor was sent for surgical pathology review (Fig. 5). The slides were stained with chromogranin, synaptophysin, hematoxylin and eosin stain revealing evidence of adrenal origin and marked catecholamine presence. The remainder of the patient’s post-operative course unveiled gradual return of vision (though not fully returned at time of discharge), hemodynamic lability and cessation of all anti-hypertensive medications upon discharge to rehab facility, and resolution of hypertensive hemolytic anemia after tumor resection. We believe this to be the first reported case in the literature of pheochromocytoma manifesting with cortical blindness secondary to presumed PRES and thrombotic microangiopathy.

## Discussion and conclusion

Pheochromocytomas theoretically should manifest with all three symptomatic manifestations of the classic Menard triad: headache, palpitations, and sweating. Nonetheless, the incidence of all three symptoms on presentation fluctuates from less than 10% up to 75% in the literature [6, 7]. With a profound increase in incidence over the past several decades, understanding the complex variability in presentation of these tumors remains paramount [8]. Prior to detailing the clinical nuances in our patient’s presentation, it is imperative to understand when clinical suspicion for pheochromocytoma should arise. This often remains quite elusive in the literature, and clinical gestalt from physicians remains the cornerstone to prompt necessary laboratory and diagnostic work-up.

Pheochromocytomas are often cited to follow the “10% rule:” 10% extra-adrenal, 10% bilateral, 10% malignant, 10% in the pediatric population, 10% having a familial component, 10% unrelated to hypertension, and 10% containing calcification [9]. Though its incidence remains identical in male patients and female patients, pheochromocytomas have been reported to present more severely in female cohorts [10]. Our case revealed a severe symptomatic presentation with multiorgan damage and to our knowledge the first-ever reported case of pheochromocytoma presenting with cortical blindness. In a remote case series conducted by Sutton et al., 75% of patients deceased with pheochromocytoma incidentally discovered at autopsy died from myocardial infarction or cerebrovascular accident [11]. Forty years later, pheochromocytoma remains underdiagnosed and underreported.

When suspecting pheochromocytoma, inherited syndromes need to be considered. Goldman et al. reported up to a quarter of pheochromocytomas have a familial component [12], with von Hippel-Lindau (VHL), multiple endocrine neoplasia 2 (MEN 2), and neurofibromatosis 1 being amongst the most common. Our patient received work-up for underlying MEN2, given this syndrome can present sporadically or be inherited. Given the potential for being malignant, it is imperative that pheochromocytoma patients have close follow-up after resection. While quoted to have 10% incidence of malignancy per the 10% rule, some reports have noted an incidence of over 20% of pheochromocytomas having malignant characteristics [13, 14]. Additionally, pathology is often indeterminate in differentiating benign from malignant, given tumor size, mitotic rate, and vascular/capsular invasion remain similar when comparing benign and malignant tumors [15].

Comprehensive laboratory biochemical testing conducted in diagnosing pheochromocytoma includes evaluation of 24-h urinary measurements of catecholamines, total and fractionated metanephrines, and VMA/HVA. Current diagnosis requires elevated plasma metanephrines or 24-h urine metanephrines; the elevation of plasma metanephrines of more than fourfold the upper limit of normal is correlated with ~ 100% probability of tumor presence [16, 17]. Once diagnosed through biochemical testing, the extent of tumor burden should be evaluated with diagnostic imaging. Computed tomography of the abdomen to initiate work-up followed by MRI with T2-weighted imaging are crucial. T2-weighted imaging on MRI reveals a “light-bulb” bright lesion (Fig. 3). MRI must be performed prior to surgery of large tumors, as in our patient, to assess vascular invasion [18]. Our patient had static CT and MR images further corroborated with functional imaging, through utilization of metaiodobenzylguanidine (MIBG) scanning. MIBG is an analog of norepinephrine with an affinity for sympathomedullary tissue, and radiotracer uptake is proportional to intralesional neurosecretory granules [19].

Upon gathering biochemical and imaging data confirming diagnosis, surgical preparation prior to adrenalectomy remains complicated and requires a multi-disciplinary approach. Adequate alpha-blockade must take place prior to surgery, as unopposed alpha-stimulation leads to vasoconstriction which can cause dangerously elevated blood pressures [20]. Given the chronicity of vasoconstriction, volume contraction and loss is commonly noted in patients with pheochromocytoma. Though there is mixed data supporting its use, implementing pre-operative saline infusion may mitigate post-operative hypotension [21, 22]. Hypoglycemia is also a possible complication to remain vigilant of post-operatively, and the use of perioperative stress-dose steroids remains controversial, given the rarity of the complication [23]. Open versus laparoscopic adrenalectomies versus robotic laparoscopic approaches depends on tumor size, anatomical variation, and surgeon preference. Hirayama et al. and others have reported the safety and feasibility of laparoscopic tumor resection, regardless of tumor size [24, 25]. Irrespective of approach used, post-operative surveillance requires intensive care monitoring immediately post-procedure, followed by serial biochemical evaluation at 1 month, 6 months, and 1 year post-operatively given the approximate 10% rate of tumor recurrence after resection [26, 27].

Given that our patient presented with concomitant cortical blindness, there are few reports in the literature of the ocular manifestations of pheochromocytoma along with its coincidence with posterior reversible encephalopathy syndrome (PRES). Bilateral blindness in the setting of pheochromocytoma has been reported manifesting as stellate neuroretinitis [28]. Han et al. presented a case of pheochromocytoma complicated by PRES in a 27-year-old male with seizures and consciousness disorder. They also noted two additional cases, one of which occurred in a pediatric patient whose initial symptoms included blurred vision and right sided paresthesias [29]. Although these cases vary slightly in presentation, especially on neurologic examination, the common link between them is the emergence of PRES preceded by an abrupt change in blood pressure to significant levels. The clinical presentation of PRES includes palpitations, anxiety, headaches, fatigue, seizures, encephalopathy, and visual disturbances [29, 30]. Characteristic radiographic findings depict cerebral vasogenic edema in the subcortical white and gray matter of the bilateral posterior parieto-occipital regions [29, 31]. MRI typically demonstrates T1 hypo- or iso- intensities, T2 & FLAIR hyperintensities in the parietal and occipital lobes, and infrequently in the cerebellum, basal ganglia, spinal cord, brain stem, centrum ovale, corpus callosum, and paraventricular area [29].

Two hypotheses have been suggested regarding the pathogenesis of PRES. One theory, “cerebral hyperperfusion,” proposes a perturbation of cerebral autoregulation triggered by severe blood pressure elevation leading to breakdown of the blood–brain-barrier (BBB) and increased permeability, the infiltration of plasma and macromolecules into brain cells, and subsequent vasogenic edema [29, 31]. The second theory, “the toxic/immunogenic theory,” suggests that exogenous (ex. chemotherapy) or endogenous (ex. sepsis) toxins disturb endothelial function eventually resulting in BBB dysfunction and cerebral edema [31]. Symptoms of PRES often overlap with those seen in pheochromocytoma. As the name implies, PRES is reversible, and if treatment is initiated immediately, patients report resolution of all symptoms with no long-term sequelae. Recommended treatment involves anti-hypertensives with a reduction in blood pressure by 20–30% within the first hours of onset [30]. Our case demonstrates how PRES can lead to considerable morbidity, given manifestation of cortical blindness in our patient. With the belief that PRES in our patient resulted from pheochromocytoma, we hope surgical resection will lead to ultimate return of vision.

In addition to manifesting concomitantly with PRES and vision loss, our patient exhibited thrombotic microangiopathy secondary to malignant hypertension. Thrombotic microangiopathy presents with thrombocytopenia and microangiopathic hemolytic anemia, and its occurrence in conjunction with a pheochromocytoma has rarely been reported [32]. Defined as systolic blood pressure more than 180 mmHg and diastolic blood pressure more than 120 mmHg with active secondary end-organ damage, hypertensive emergency leads to rapid decompensation with potentially fatal consequences if left untreated. In our case, uncontrolled arterial pressure resulted in endothelial injury precipitating platelet and erythrocyte destruction. This can lead to further vascular damage, as overt sympathetic activation triggers elevated plasma renin activity, leading to a cascade that may eventually result in renal failure [33]. Evidenced through thrombocytopenia, elevated lactate dehydrogenase, and schistocytosis on blood smear, we ultimately determined our patient to harbor the diagnosis of thrombotic microangiopathy. Left undetected, this complication alone can lead to significant morbidity and mortality. Therefore, it is crucial to include pheochromocytoma as the precipitant to resistant hypertension in patients with previously well-controlled blood pressure, who present with thrombotic microangiopathy. Pathological evolution to renal failure demands complex management, often mandating hemodialysis with transfusions of multiple blood products. Our patient’s renal failure was caught early in her course, and aggressive medical management prevented potentially catastrophic renovascular compromise [34].

The significant variability in the clinical presentation of a pheochromocytoma should leave it on the differential diagnosis of all clinicians. Herein, we reported a unique manifestation of this tumor in the setting of hypertensive emergency precipitating posterior reversible encephalopathy syndrome, leading to cortical blindness and thrombotic microangiopathy causing acute kidney injury. We hope our case contributes an additional vignette to the ever-growing literature revolving adrenal tumors, their symptomatic presentation, and their complex management. We also hope this report prompts increased judicious suspicion amongst diagnosticians when evaluating patients with refractory hypertension.

## Data Availability

All the data generated and/or analyzed during this study are included in this published article.

## Abbreviations

PRES: Posterior Reversible Encephalopathy Syndrome
TMA: Thrombotic Microangiopathy
CT: Computed Tomography
CTA: Computed Tomography with Angiography
MRI: Magnetic Resonance Imaging
VMA: Vanillylmandelic Acid
HVA: Homovanillic Acid
MIBG: Iodine-123 meta-iodobenzylguanidine
MEN2A: Multiple endocrine neoplasia syndrome type 2A
CKD: Chronic Kidney Disease
AKI: Acute kidney injury
